# Synthesis, Characterization and Biological Activities of New Schiff Base Compound and Its Lanthanide Complexes

**DOI:** 10.3390/ph15040454

**Published:** 2022-04-07

**Authors:** Abdel-Aziz Abu-Yamin, Maisa Siddiq Abduh, Sultan Ayesh Mohammed Saghir, Naif Al-Gabri

**Affiliations:** 1 Department of Chemistry, Al-Hussein Bin Talal University, Ma’an 71111, Jordan; 2 Department of Medical Laboratory Sciences, Faculty of Applied Medical Sciences, King Abdulaziz University, Jeddah 21589, Saudi Arabia; 3 Center of Excellence in Genomic Medicine Research, King Abdulaziz University, Jeddah 22252, Saudi Arabia; 4 Department of Medical Analysis, Princess Aisha Bint Al-Hussein College of Nursing and Medical Sciences, Al-Hussein Bin Talal University, Ma’an 71111, Jordan; sultan.s.ayesh@ahu.edu.jo; 5 Veterinary Department, Faculty of Agriculture and Veterinary Medicine, Thamar University, Dhamar 2153, Yemen; naifaljabry2014@gmail.com; 6 Laboratory of Salam Veterinary Group, Al-Qassim, Buraydah 51911, Saudi Arabia

**Keywords:** synthesis, spectroscopic, 2-Amino-6-ethoxybenzothiazole, 3-(2-Furyl)acrolein, lanthanide complexes, antimicrobial, antitumor, antioxidant

## Abstract

The thermal condensation of 3-(2-Furyl)acrolein with 2-Amino-6-ethoxybenzothiazole generated a new Schiff base, (1E,2E)-N-(6-ethoxybenzo[d]thiazol-2-yl)-3-(furan-2-yl)prop-2-en-1-imine (**L**), with general formula of C_16_H_14_N_2_O_2_S. Also, a series of lanthanide complexes of gadolinium, samarium, and neodymium (**La**–**Lc**) were synthesized utilizing acetonitrile as the solvent and triethylamine as a buffer and catalyst. Based on elemental analysis, mass spectroscopy, and FTIR analysis, all of the Bis-(1E,2E)-N-(6-ethoxybenzo[d]thiazol-2-yl)-3-(furan-2-yl)prop-2-en-1-iminetri-nitratolanthanide(III) complexes with the general formula [Ln**L**_2_(NO_3_)_3_]·H_2_O are solids with a 2:1 molar ratio (ligand: metal). Based on conductivity estimates, they are nonelectrolytes and monoatomic paramagnetic according to the magnetic moment measurements, and one mole of lattice water was found after thermal gravimetric measurements and FTIR analysis. Therefore, the lanthanide complexes show a ten-coordination structure with a deformed bicapped square antiprismatic. The Schiff base and its complexes were screened for their antimicrobial, antifungal, antioxidant, and antitumor properties. Their antimicrobial and antifungal activities were strong, and they also produced good antioxidant and antitumor effects.

## 1. Introduction

Schiff base compounds are compounds with an azomethine group that arise as the result of a reversible acid-catalyzed condensation reaction between primary amine and carbonyl compounds, as described by Hugo Schiff in 1864 [1,2]. Schiff base compounds are an important and extensively investigated class due to their vast range of biological applications, ease of production, chelating characteristics, and stability [3,4,5]. There are many different kinds of lanthanides (Ln-L) that behave differently with bacteria, depending on the kind of bacteria and type of lanthanide [6]. Additionally, (Ln-L) have a variety of useful applications in physics, including luminescence, as well as in solar cells [7,8]. The compound 3-(2-Furyl)acrolein can be prepared by several methods, such as via Witting [9], Heck [10], and Stille reactions [11]. Benzothiazole is a privileged bicyclic ring system that is composed of a benzene ring fused to a thiazole ring; one of its derivatives, 2-Amino-6-ethoxybenzothiazole, acts as a neuroprotective agent in a variety of animal models of brain illness [12].

Benzothiazole can be derived by forming complexes with azomethine (CH=N) and thiazole (C=N) rings, and they form complexes with azomethine and sulfur in thiazole rings. The lanthanide complexes in the following work followed the second route, as evidenced from the spectral and analytical data [13].

Several biological activities of Schiff base compounds have been documented in the literature, including antibacterial, antifungal, anti-inflammatory, and anticancer properties [14,15,16,17,18,19]. Hence, in the present study, a new Schiff base derived from 3-(2-Furyl)acrolein with 2-Amino-6-ethoxybenzothiazole was synthesized, and its complexes with gadolinium, samarium, and neodymium were prepared using the imine nitrogen and sulfur of the thiazole ring and were investigated through several physicochemical analyses. It will be feasible to investigate newly synthesized Schiff base compounds and **La**–**Lc** as dyes in the future, particularly because they have a unique shiny orange hue. In addition, it would be interesting to investigate the biological activities of this new Schiff base compound and its lanthanide complexes, such as their antibacterial, antifungal, antioxidant, and antitumor properties.

## 2. Results and Discussion

### 2.1. Synthesis

**Scheme 1:**
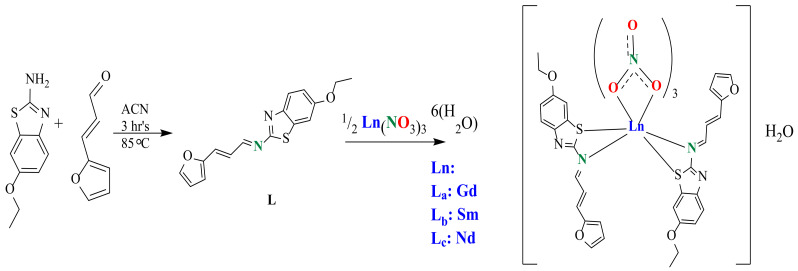
Synthesis of Schiff base **L** and its Ln^III^ complexes (**La**–**Lc**).

A new Schiff base (**L**), C_16_H_14_N_2_O_2_S, was obtained from the condensation of 3-(2-Furyl)acrolein with 2-Amino-6-ethoxybenzothiazole in ethanolic solution; their coordination complexes [Ln**L**_2_(NO_3_)_3_]·H_2_O (**La**, **Lb**, and **Lc**) were accessible from the reaction of [Ln(NO_3_)_3_·6H_2_O] (Ln; a = Gd; b = Sm and c = Nd) with **L** in 1:2 molar ratios in refluxing acetonitrile (Scheme 1). After cooling the reaction mixture to room temperature and after allowing the reaction solvent to evaporate at room temperature, the solid precipitate that had formed was filtered off and washed with cold ethanol and diethyl ether (see experimental section). Analytically pure samples were checked by means of melting point measurements. Complexes **La**, **Lb**, and **Lc** were stable in moisture and air as well as in solution and in the solid state. According to the lanthanide complex binding rule, only six lanthanide(III) ion coordination sites can be filled with oxygen atoms from nitrate anions, whereas four can be filled with nitrogen and sulfur atoms from two bidentate ligands to generate a ten-coordination number (bicapped square antiprismatic) [20]. Water molecules are not included in the inner coordination sphere, but mass spectroscopy, FTIR, and elemental analysis revealed that the complexes contain a 1:2 (lanthanides: ligand) stoichiometry, so the crystalline water is crystallized outside of the coordination sphere. The chemical structure of the ligand (**L**) was confirmed by the ^1^H-NMR and ^13^C-NMR spectra, but this was not possible for the complexes due to the paramagnetic nature of the complexes. Other methods were used to infer the structure of the new Schiff base and its complexes, including FTIR, mass spectrometry, chemical analysis, magnetic susceptibility investigations, and conductivity tests. According to the molar conductance values obtained in isopropanol solutions (1.0 mM), all of the complexes had molar conductance ranging from 23 to 28 S·cm^2^mol^−1^, indicating that they are non-electrolytic in nature [21]. All of the complexes were insoluble in nonpolar solvents, whereas they were all extremely soluble polar solvents such as methanol, isopropanol, acetone, toluene, and acetonitrile as well as DMSO. The magnetic moment values show that the tri-positive lanthanide ions are paramagnetic due to the presence of unpaired 4f electrons, which are effectively shielded by 5 S^2^ and 5 p^6^ electrons [22]; this suggests that all complexes exhibit mononuclear trivalence [23].

### 2.2. Spectral Investigations of the Ligand (L) Using ^1^H-NMR and ^13^C-NMR

The ^1^H-NMR spectrum data of Schiff base **L** in acetonitrile solvent revealed seven signals, the most significant of which was at 8.68 ppm, which corresponds to the azomethine proton [24], as well as a signal at 164.0 ppm in the ^13^C-NMR spectrum, which corresponds to the carbon of the same group, as shown in Figures S1 and S2 (see SI file). The additional signals are depicted in the diagrams. Finally, because of their paramagnetic properties, NMR investigations of the lanthanide compounds proved to be impossible.

### 2.3. FTIR Spectral Investigations of Schiff Base ***L*** and Related Ln^III^ Complexes (***La***–***Lc***)

The Fourier Transform Infrared (FTIR) spectra of the ligand and its lanthanide complexes are presented in Figure S3. The initial identification of **L** was made on the basis of the absence of the IR bands corresponding to the amino group of 2-Amino-6-ethoxybenzo thiazole and the aldehyde group of 3-(2-Furyl)acrolein. This was confirmed by the appearance of new bands at 1660 and 1016 cm^−1^ corresponding to the υCH=N and γCH=N of the azomethine group [25] and by the bands at 1603 and 758 cm^−1^ corresponding to the υC=N and υC–S–C of the thiazole ring [26,27]. According to the spectral data in Table 1, the observed shift in the peak positions in the complex FTIR spectra indicates that though complexation, the nitrogen atom of the azomethine group and the sulfur atom of the thiazole ring moiety are involved in the complexation [25]. In addition, new bands were observed for Ln-N at 547, 563, and 558 cm^−1^ for all of the complexes and for Ln-S at 416, 428, and 421 cm^−1^, respectively, for **La**, **Lb**, and **Lc** [28].

The existence of ionic nitrate in the complexes was suggested by the presence of nitrate groups attached to the Ln^III^ ion in a bidentate manner at 1394 cm^−1^ [29]. To validate the lattice water molecules with ionic lanthanides, wide bands in the spectra centered at 3450, 3477, and 3463 cm^−1^ emerged in **La**, **Lb**, and **Lc**, respectively, [30] a finding supported by TGA analysis.

Complexation had no effect on several bands, such as those in the furan ring and alkene chain, resulting in the following bands: absorption at 993 cm^−1^, which is the out-of-plane =C–H bending mode of the trans di-substituted alkene [31] as well as at 1572 cm^−1^ (furan ring with –C=C–), at 1215 cm^−1^ (furan ring with –C–O–) [32], and at 1057 cm^−1^, all of which are the result of aromatic C–O stretching (furan) [32]; at 3107 cm^−1^ as a result of =C–H aromatic stretching (furan ring) [33]; at 822 cm^−1^ due to C–H alkene bending (furan) [33]; and at 1460, 1452, 1455 and 1461 cm^−1^ due to Ar. C=C for **L**, **La**, **Lb**, and **Lc**, respectively [34]. C–N bonds are observed at 1310 cm^−1^ [35]. Peaks at 2972 cm^−1^ (asymmetric stretch vibration of C–H of CH_3_), 2867 cm^−1^ (υ of CH_3_), 1464 cm^−1^ (asymmetric bending of CH_3_), and 1387 cm^−1^ (in-plane asymmetric CH_3_ bending) [36] confirm the presence of a methyl group in the alkyl portion in addition to the aliphatic ν(C–H) at 2905 cm^−1^, 1124 cm^−1^, and 1188 cm^−1^ [37]. The strong sharp bands at 760 cm^−1^, 793 cm^−1^, and 822 cm^−1^ can be ascribed to o-disubstituted benzene ring vibration [38].

The two bands at 3107, 3103, 3085, and 3100 cm^−1^ and at 2979, 2984, 2974, and 2986 cm^−1^ are due to the asymmetric and symmetric stretching vibrations of the CH group of the aromatic ring for **L**, **La**, **Lb**, and **Lc** respectively.

Finally, the ATR was used to confirm the FTIR spectrum of complexes rather than the KBr technique since bromide replaces coordinated nitrate in the spectrum and disappears from the spectrum [39].

### 2.4. UV-Vis Absorption Spectra

Figure S4 displays the electron absorption spectra of **L**, **La**, **Lb**, and **Lc** in the wavelength range of 200–800 nm in acetonitrile solvent (see the ESI). Strong bands are visible in the 350–500 nm region, with a maximum lambda of 359 nm corresponding to the n-π * of the ligand **L**, [40], while the band at λmax 310 nm corresponds to the π-π * transitions of **L** [41]. The first band exhibits a slight red shift of 3–7 ppm after complexation with gadolinium, samarium, and neodymium, whereas the second band exhibits a slight red shift of 9–11 ppm, suggesting that the nitrogen atom from the imine is involved in the complexation, as verified by FTIR analysis.

**Figure 1:**
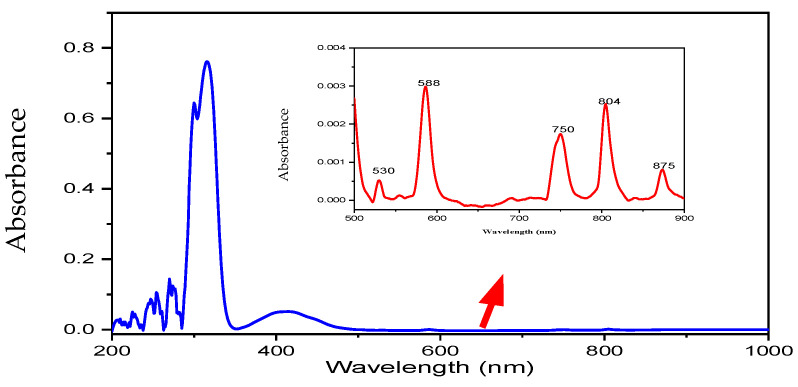
UV–visible spectra of concentrated DMF solution of **Lc**.

Figure 1 displays the UV-vis spectrum of the **Lc** complex, which features distinctive parity-forbidden narrow 4f–4f absorption bands originating from the ^4^I_9/2_ ground state to different excited states of the Nd^+3^ ion (1.0 × 10^−2^ M in dimethylformamide, DMF). The spectrum is expanded from 500 to 900 nm to illustrate the transitions that were found; the reported transitions are as follows: ^4^F_3/2_ ← ^4^I_9/2_ (875 nm); ^4^F_5/2_, ^2^H_9/2_ ← ^4^I_9/2_ (804 nm); ^4^S_3/2_, ^4^F_7/2_ ← ^4^I_9/2_ (750 nm); ^4^G_5/2_, ^2^G_7/2_ ← ^4^I_9/2_ (588 nm); and ^4^G_9/2_, ^4^G_7/2_, ^2^K_13/2_ ← ^4^I_9/2_ (530 nm). In the visible area, the transition ^4^I_9/2_ → ^4^G_5/2_ at 588 nm represents a hypersensitive transition, whereas the less intense transition ^4^I_9/2_ → ^2^G_7/2_ was an overlap [42,43,44].

### 2.5. Mass Spectra

Mass spectra provide vital clues for elucidating the structure of compounds. The mass spectra of the ligand **L** and its lanthanide complexes [Ln**L**_2_(NO_3_)_3_]·H_2_O were recorded and are shown in Figures S5–S8, where their molecular ion peaks agree with the calculated formulae. The molecular ion peak for the ligand **L** is observed at 298.80 *m*/*z* (calcd. 298.36) with 26.90% abundance, which is associated with the molecular ion, C_16_H_14_N_2_O_2_S^+^, whereas its gadolinium complex shows the molecular ion peak at 958.77 *m*/*z* (calcd. 957.997) with 39.88% abundance, which is related to its molecular ion [Gd**L**_2_(NO_3_)_3_]·H_2_O, which confirms the **La** stoichiometry of 2 ligand: 1 metal. The samarium complex shows the molecular ion peak at 952.63 *m*/*z* (calcd. 951.107) with 25.81% abundance, which is related with its molecular ion [Sm**L**_2_(NO_3_)_3_]·H_2_O, which confirms the **Lb** stoichiometry of 2 ligand: 1 metal, while the neodymium complex shows the molecular ion peak at 947.69 *m*/*z* (calcd. 944.989) with 21.80% abundance, which is related to its molecular ion [Nd**L**_2_(NO_3_)_3_]·H_2_O, which confirms the **Lc** stoichiometry of 2 ligand: 1 metal. The elemental analysis values shown in Table 2 further support the results of the mass studies, as they are in good agreement with the values calculated from the molecular formula assigned to these complexes.

### 2.6. Thermogravimetric Analysis (TGA)

Thermogravimetric analysis was used to compute the water molecules within or outside the inner coordination sphere as well as any coordinated or crystalline water molecules and to estimate the thermal stability of the novel metal complexes.

**Figure 2:**
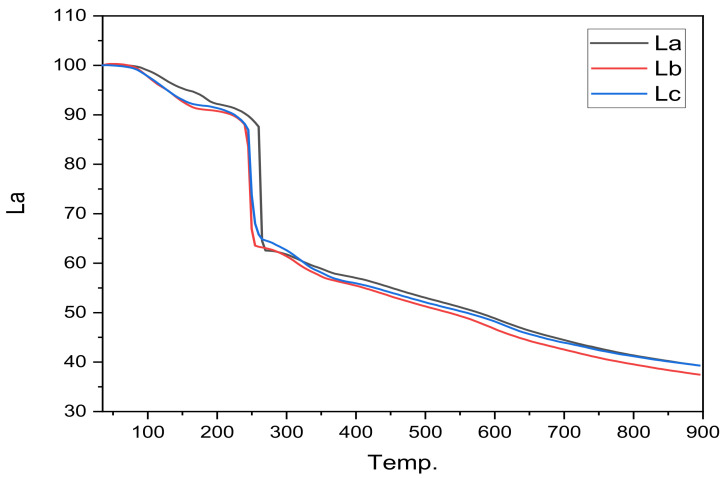
TGA thermograms of the three complexes: **La**, **Lb**, and **Lc**.

The TGA thermograms of [Ln**L**_2_(NO_3_)_3_]·H_2_O, as shown in Figure 2, were adequately regulated at a current of 10 °C min^−1^ under an N_2_ atmosphere in the temperature range of 50 °C to 800 °C and at a heating rate of 20 °K·min^−1^. The thermogravimetric spectra of the complexes show different breakdown stages, with a progressive weight loss step being shown in detail in Figures S9–S11 (see the ESI). Figure S9 for **La** that has four stages; the first stage indicates that the complex has one mole of lattice water by losing 1.90% of its weight (clad. 1.89%) in the temperature range of 35–115 °C; the second stage takes place within the temperature range of 115–226 °C, with the 6.41% (clad. 6.50%) loss in weight corresponding to the NO_3_ molecule; the weight loss in the third stage loss 31.10% (clad. 31.20%) and takes place within the temperature range of 226–293 °C and corresponds to one mole of the ligand **L** molecule [45]. Finally, the gradual curvature was expected to reach 39.33% (cald. 38.10%) residue, representing gadolinium (III) oxide.

In the same manner, **Lb**, shown in Figure S10, (see the ESI), includes four stages; the first stage indicates that the complex has one mole of lattice water via a 1.90% (cald. 1.90%) loss in the temperature range of 35–100 °C; the second stage takes place within the temperature range of 100–188 °C, with a 6.60% (cald. 6.52%) weight loss corresponding to NO_3_ molecule; the third stage loss of 29.50% (cald. 31.40%) takes place within the temperature range of 188–259 °C and corresponds to one mole of the ligand **L** molecule [46]. Finally, the gradual curvature was expected to reach 37.46% (cald. 36.77%) residue, representing samarium (III) oxide.

Similarly, **Lc,** shown in Figure S11, (see the ESI), includes four stages; the first one includes a 1.90% loss (cald. 1.90%) at the temperature range of 35–102 °C, corresponding to one mole of lattice water; the second stage takes place within the temperature range of 102–208 °C, with a 6.45% (cald. 6.57%) weight loss corresponding to the NO_3_ molecule; the third stage loss of 30.10% (cald. 31.55%) takes place within the temperature range of 208–283 °C and corresponds to one mole of the ligand **L** molecule [36]. Finally, the gradual curvature was expected to reach 37.40% (cald. 35.60%) residue, representing Neodymium (III) oxide.

### 2.7. Antioxidant Activity

In our bodies, reactive oxygen species such as superoxide anion, hydroxl radicals, and hydrogen peroxide ROS can form as a result of metabolic reactions. These ROS are considered to be highly reactive and potentially damaging substances [18,47]. A variety of chronic diseases such as heart disease, cancer, and aging can occur due to the oxidative damage that ROS causes in lipids, proteins, and nucleic acids [48]. It is therefore important to administer pharmaceuticals that are rich in antioxidants to prevent free radical damage in the body [18]. In our antioxidant assays for the Schiff base compound and it complexes **La**, **Lb**, and **Lc**, we used TAC and DPPH to evaluate their antioxidant activity.

#### 2.7.1. Total Antioxidant Activity

The TAC of the newly synthesized Schiff base compound and its complexes showed different antioxidant activities that were expressed as a mg gallic acid equivalent per gram sample (mg GAE/g), as shown in Table 3. The free ligand **L** and its lanthanide complex **Lc** showed the best antioxidant activity (25.97 and 28.85 mg GAE/g, respectively) compared to ascorbic acid, which was used as a standard (66.08 mg GAE/g). The TAC of the free ligand **L** and its complex **Lc** constitute about 39.30 and 43.65%, respectively, compared to ascorbic acid, which was used as a standard. Based on these findings, we were able to assess the total antioxidant activity of the studied complexes as follows: **Lc** > **L** > **La** > **Lb**, and the results are clearly presented in Table 3. In the current study, the total antioxidant capacity of the newly synthesized Schiff base compound and its complexes was comparable to the results of the other Schiff base compounds and their complexes from previous studies [18,49].

#### 2.7.2. DPPH Radical Scavenging Activity

In contrast, the weak radical scavenging activity of the Schiff base compound and its complexes was detected using the DPPH assay and though comparisons with standard ascorbic acid (IC_50_ = 10.56) (Table 3). Previous studies that have evaluated the radical scavenging activity of Schiff base compounds and their complexes have found similar low DPPH values as ours. [17]. Other studies showed different results with high DPPH scavenging activity compared to the findings of the present study [18,49,50,51].

### 2.8. Biological Activities

#### 2.8.1. Antimicrobial and Antifungal Activity

The antimicrobial and antifungal activity of the tested microorganisms were expressed as the minimum inhibitory concentration (MIC) in mm and presented as mean ± SD (Table 4). The antimicrobial activity of the free ligand and its complexes were explored using two strains of Gram-positive bacteria (*Staphylococcus aureus* (ATCC 25923) and *Bacillus subtilis* (RCMB 015)) and two strains of Gram-negative bacteria (*Escherichia coli* (ATCC 25922) and *Proteus vulgaris* (RCMB 004)). The results showed that the **Lc** complex had higher antibacterial activity against both Gram-positive (18.07–20.80 mm) and Gram-negative (18.60–22.60 mm) bacteria compared to the free ligand **L** and the other complexes, but its activity was lower than that of conventional medication (gentamycin (24.01–29.90 mm)) (Table 4). The results of metal complex **Lc**, which showed higher activity over the free ligand, and the lipophilicity of the metal ions in the metal chelates explains such enhanced activity (Table 4). The study also suggests that the complexes possess antibacterial activity that inhibits the growth of microbes by blocking their active mechanisms [52]. Compared to previous studies, the present study showed similar results in terms of the antimicrobial activity of the free ligand and its complexes [18,52,53].

On the other hand, the antifungal activity of the free ligand **L** and its complexes **La**–**Lc** was shown to be higher than that of the control during antifungal investigations employing *Aspergillus fumigatus* (RCMB 002008) (Ketoconazole). The free ligand and its complexes demonstrated substantial antifungal activity against *Candida albicans* (RCMB 005003), with the free ligand exhibiting the strongest activity compared to the standard drug. Jarrahpour and his colleagues reported that the free ligand and its complexes had strong antifungal activity in their studies as well [52].

Metal complexes have been demonstrated to have very strong antibacterial activity, which can be attributed to their high lipophilic nature [54]. According to the cell permeability idea, the lipid membrane that surrounds the cell only allows lipid-soluble molecules to pass through; hence, liposolubility was thought to be a key factor in antimicrobial activity management [54]. According to the chelation process, the large reduction in metal ion polarity was caused by orbital overlap in the ligand and the partial sharing of the metal ion’s positive charge with donor groups.

Additionally, it improved the lipophilicity of the complex by increasing electron delocalization across the entire chelate ring. The penetration of complexes into the lipid membrane will increase as a result of this increase in lipophilicity, and the metal binding sites on the microbial enzymes will be blocked. These metal complexes also have the ability to disrupt the cell’s respiration mechanism, leading to protein synthesis being hampered and the organism’s growth to be delayed. Conductivity, solubility, and the length of the connection between the metal and the ligand are all factors that can increase activity [55,56]. The results of the current study on the antimicrobial activities of this new Schiff base compound and its metal derivatives are similar to the findings of previous studies by Elsonbati et al., 2021, which demonstrated that these compounds had strong antimicrobial activity [57].

#### 2.8.2. Cytotoxic Activity

The cytotoxic effects of the newly synthesized Schiff base compound and its complexes against several cell lines are presented as the calculated IC_50_ values of the tested compounds and are shown in Table 5.

The findings of the present study exhibit that the free ligand **L** has a moderate cytotoxic effect against human hepatocellular carcinoma (HepG-2) (IC_50_ = 41.19 μg/mL), and weak cytotoxic effects were detected against prostate carcinoma (PC-3) and human breast cancer cells (MCF-7) (IC_50_ = 58.81 and 84.90 μg/mL, respectively), and no effects were observed against normal liver human amnion cells (WISH) (IC_50_ > 500 μg/mL). Compound **La** presented weak activity against lung carcinoma (A-549) (IC50 = 83.23 μg/mL), whereas no effects were recorded against the other cell lines (human hepatocellular cancer cell (Huh-7), human breast carcinoma (MDA-MB231), and normal human lung fibroblast (MRC-5) (IC_50_ = 164.72, 106.31, and >500 μg/mL, respectively)). In addition, compound **Lb** displayed moderate activity against human colon carcinoma (HCT-116) and normal human lung fibroblast cells (CACO2) (IC_50_ = 50.21 and 40.15 μg/mL, respectively), but weak activity was detected against human muscle rhabdomyosarcoma cells (IC_50_ = 82.52 μg/mL), and no activity was noticed against normal human lung fibroblast cells (WI-38) (IC_50_ ≥ 500). Furthermore, compound **Lc** displayed moderate activity against human hepatocellular carcinoma (HepG-2) (IC_50_ = 42.18 μg/mL), and weak activity was detected against human breast cancer cells (MCF-7) (IC_50_ = 55.42 μg/mL), while no activity was observed against larynx carcinoma cells (HEP-2) and against normal human melanocytes (HFB4) (IC_50_ = 105.23 and >500 μg/mL, respectively) (Table 5). In the current study, based on the IC_50_ values of the compound, we used the following grades to classify the degree of the cytotoxic activity as follows: IC_50_ (μg/mL): 1–10 (very strong), 11–20 (strong), 21–50 (moderate), 51–100 (weak), and above 100 (non-cytotoxic) [17]. According to the results of the present study, the newly developed Schiff base compound and its complexes display cytotoxicity that is consistent with previous studies that showed a wide range of cytotoxicities [46,48].

## 3. Materials and Methods

### 3.1. Material and Measurements

Sigma-Aldrich (St. Louis, MO, USA), TEDIA (Fairfield, OH, USA), and Merck (Darmstadt, Germany) provided all of the chemicals. A Bruker-Alpha II spectrometer (Bruker Optik GmbH, Ettlingen, Germany) was used to capture the FT-IR spectra using the ATR technique in the mid-infrared region (400–4000 cm^−1^) with a 4 cm^−1^ resolution and 24 scans. Thermogravimetric analysis (TGA) was used to investigate the thermal behavior of all of the complexes in a nitrogen environment (N_2_) at a heating rate of 10 °C/min using a NETZSCH TG 209 f3 (NETZSCH Scientific Instruments Co., Selb, Germany). The ^1^H and ^13^C NMR spectra in DMSO solvent were recorded using a Bruker Avances III-500 MHz. An SMP 10 electrothermal melting point device was used to determine the melting points. A CP-411 Handheld Waterproof pH-mV conductometer (Elmetron, Zabrze, Poland) was used to measure the conductance in methanol (1.0 mM) at room temperature. The elemental contents of the synthesized compounds were determined using a FLASH 2000 CHNS/O analyzer, Thermo Scientific, Waltham, MA, USA. Mass spectra performed using the direct inlet tool in the Thermo Scientific GCMS ISQ model. The UV-visible spectrum was collected using a SPECORD PLUS–Analytik Jena AG (Jena, Germany). At ambient conditions, the electronic absorption spectra of all of the compounds in acetonitrile at 1.0 mM were measured in the 200 to 800 nm range. Magnetic susceptibility values of the prepared complexes were determined using the Johnson Matthey Magnetic Susceptibility Balance Model Mkic = 0.949. The purity of the substances was validated using thin-layer chromatography.

### 3.2. Synthesis of Schiff Base I, C_16_H_14_N_2_O_2_S

The new Schiff base (**L**) was made using the method described in [58]: 0.482 g/ 3.95 mmol of 3-(2-Furyl)acrolein was dissolved in 10 mL of ethanol. The solution was stirred for 15 min at room temperature followed by the addition of 15 mL of an ethanolic solution with an equimolar amount of 2-Amino-6-ethoxybenzothiazole (0.767 g, 3.95 mmol); the solution was then refluxed at 85 °C for 3 h, and the color changed to yellow-brown after cooling down. After that, the solution was allowed to precipitate at room temperature for 24 h, yielding an orange-yellow precipitate: (1E,2E)-N-(6-ethoxybenzo[d]thiazol-2-yl)-3-(furan-2-yl)prop-2-en-1-imine (I). Table 2 lists all of the physicochemical characteristics. ^1^H NMR (500 MHz, acetonitrile-d3): δ 8.68 (d, H1), δ 7.73 (d, H2), δ 7.29 (d, H3), δ 7.17 (d, H4), 6.82 (d, H5), 6.57 (d, H6), 5.89 (t, H7), 3.98 (q, H8), 1.32 (t, H9; 13C NMR (126 MHz, CD_3_CN): δ 167.14, 165.47, 155.37, 147.47, 146.70, 135.26, 133.43, 124.18, 119.72, 118.15, 116.77, 116.36, 114.61, 113.82, 106.16, 64.78, and 15.03. FTIR (ATR, cm^−1^): 1624, 1600, 647, 1057. UV/vis (λ_max_): 310 nm, 359 nm. MS (positive ion mode) *m*/*z*: Calcd. for C_16_H_14_N_2_O_2_S (M+H)+ to be 298.4 but found to be 298.80.

### 3.3. Synthesis of Lanthanide Schiff Base Complexes **La**, **Lb** and **Lc**; Ln: Gd, Sm and Nd Respectively

The compound (1E,2E)-N-(6-ethoxybenzo[d]thiazol-2-yl)-3-(furan-2-yl)prop-2-en-1-imine (**L**) (500 mg, 1.68 mmol) was dissolved in 15 mL of acetonitrile, and a solution of [Ln(NO_3_)_3_·6H_2_O] (0.838 mmol) in 15 mL of acetonitrile was added dropwise over 10 min at ambient temperature. The resulting reaction mixture was refluxed for 3 h and was then evaporated at room temperature for one week to create pale orange-colored crystals. After filtration, the solid obtained was washed with cold ethanol (3 × 5 mL) and 5 mL diethyl ether. According to the following equation, (μ_eff_ = g[S(S+1)]½ = 7.94 μB), the effective magnetic moment for **La** was 7.90 μB, which is almost the same as the spin-only value expected for the free ion [19], while for **Lb**, it was 1.88 μB, very close to 1.7 μB, and for **Lc,** it was 3.4 μB, close to 3.5 μB [20]. Table 2 details all of the physicochemical data for the Schiff base compound (**L**) and its complexes **La**, **Lb**, and **Lc**.

### 3.4. Antioxidant Activities

#### 3.4.1. Total Antioxidant Capacity Assay

The total antioxidant capacity (TAC) of the newly synthesized Schiff base compound and its complexes was evaluated by means of the phosphomolybdate complex method proposed by Prieto et al. [59].

A 0.2 mL amount of methanol sample solution was mixed with 0.1 mL of reagent solution (0.6 M sulphuric acid, 2 mM sodium phosphate, and 4 mM ammonium molybdate). The mixture was incubated at 95 °C for 90 min in a thermal block. The samples were cooled, and their absorbances were read at 695 nm. TAC was measured in triplicate as mg gallic acid equivalents per gram sample (mg GAE/g). Butyl hydroxytoluene (BHT) was used as the standard.

#### 3.4.2. DPPH Radical Scavenging Activity Assay

A freshly prepared (0.004% *w*/*v*) methanol solution of 2,2-diphenyl-1-picrylhydrazyl (DPPH) radical was prepared and stored at 10 °C in the dark. A methanol solution of the test compound was prepared. A 40 µL aliquot of the methanol solution was added to 3 mL of DPPH solution. Absorbance measurements were recorded immediately with a UV-visible spectrophotometer (Milton Roy, Spectronic 1201, Houston, TX, USA). The decrease in the absorbance at 515 nm was determined continuously, with data being recorded at 1 min intervals until the absorbance stabilized (16 min). The absorbances of the DPPH radicals without the antioxidant (control) and of the reference compound, ascorbic acid, were also measured. All of the determinations were performed in triplicate. The percentage of inhibition (PI) of the DPPH radical was calculated according to the following formula:

% Inhibition = (AC−AS)/AC × 100,

where AC is the absorbance of the control, and AS is the absorbance of sample [55,60].

The 50% inhibitory concentration (IC_50_) is defined as the concentration required for 50% DPPH radical scavenging activity and was estimated from graphic plots of the dose–response curves using Graphpad Prism software (San Diego, CA, USA). Vitamin c (ascorbic acid) was used as the standard.

### 3.5. Biological Activities Assessment

#### 3.5.1. Antimicrobial and Antifungal Activity Assessment

The antimicrobial activity was investigated on the newly synthesized Schiff base compound and its complexes in order to increase the selectivity of these derivatives towards test microorganisms.

The antimicrobial profile was tested against two species of bacteria, Gram-positive bacterial species (*Staphylococcus aureus* and *Bacillus subtilis*) and Gram-negative bacterial species (*Escherichia coli*, *Proteus vulgaris*), as well as against fungi, including one filamentous fungus (*Aspergillus fumigatus*) and one yeast species (*Candida albicans*) using a modified well-diffusion method. Briefly, 100 μL of the test bacteria/fungi were grown in 10 mL of fresh media until they reached a count of approximately 108 cells/mL for bacteria or 105 cells/mL for fungi. A 100 μL amount of microbial suspension was spread onto agar plates corresponding to the broth in which they were maintained and tested for susceptibility using the well-diffusion method. A 100 µL amount of each sample (at 10 mg/mL) was added to each well (6 mm diameter holes cut into the agar gel). The plates were incubated for 24–48 h at 37 °C (for bacteria and yeast) and for 48 h at 28 °C (for filamentous fungi). After this incubation period, the microorganism’s growth was observed. The resulting inhibition zone diameters were measured in millimeters and used as a criterion for the antimicrobial activity. If an organism is placed on the aga and then does not grow in the area around the well, then it is susceptible to the chemical. This no-growth area around the disc is known as a “zone of inhibition” or “clear zone”. The size of the clear zone is proportional to the inhibitory action of the compound under investigation. Solvent controls (DMSO) were included as negative controls in every experiment. DMSO was used to dissolve the tested compound and showed no inhibition zones, confirming that it has no influence on the growth of the tested microorganisms [56,61].

Tests were also performed on positive controls, and gentamycin was used a as standard antibacterial drug, and ketoconazole was used as a standard antifungal drug [62]. All of the biologically active samples were tested to determine the MIC using the broth microdilution method. After incubation, the lowest concentration showing complete growth inhibition was recorded as the MIC of the respective sample.

#### 3.5.2. Antitumor Activity Assessment

##### Cell Culture and Treatment

The cells were propagated in Dulbecco’s modified Eagle’s medium (DMEM) supplemented with 10% heat-inactivated fetal bovine serum, 1% L-glutamine, HEPES buffer, and 50 µg/mL gentamycin. All of the cells were maintained at 37 °C in a humidified atmosphere with 5% CO_2_ and were sub-cultured two times a week [63].

##### In Vitro Cytotoxicity Assessment

For the cytotoxicity assay, we have used different types of cell lines which include MCF-7 (ATCC No. HTB-22) human breast cancer cell line, HepG-2 cells (ATCC No. HB-8064) human Hepatocellular carcinoma cell, HCT-116 cells (ATCC No. CCL-247) human colon carcinoma cell, MDA-MB-361 cells (ATCC No. HTB-27) human breast cancer cell, A549 cells (ATCC No. CCL-185) human lung cancer cell, PC-3 cells (ATCC No. CRL-1435) human prostate cancer cell, Huh-7 cells (ATCC No. CVCL-0336) human Hepatocellular carcinoma cell, MRC-5 cells (ATCC No. CCL-171) Normal human lung fibroblast cell, WI-38 cells (ATCC No. CCL-75) Normal human lung fibroblast cell, RD cells (ATCC No. CRL-136) human prostate cancer cell, HEp-2 cells (ATCC No. CCL-23) human epidermoid carcinoma of the larynx cell, CACO2 cells (ATCC No. HTB-37) human colorectal adenocarcinoma isolated from Large intestine and WISH cells (ATCC No. CCL-25) human epithelial amnion normal Liver cell. All the cell lines were purchased from American Type Culture Collection (ATCC; Rockville, MD, USA).

The cells were seeded in 96-well plates at a cell concentration of 1 × 104 cells per well in 100 µL of growth medium. Fresh medium containing different concentrations of the test sample was added after 24 h of seeding. Serial two-fold dilutions of the tested chemical compound were added to confluent cell monolayers dispensed into 96-well, flat-bottomed microtiter plates (Falcon, Franklin Lakes, NJ, USA) using a multichannel pipette. The microtiter plates were incubated at 37 °C in a humidified incubator with 5% CO2 for a period of 24 h. Each concentration of the test sample was tested in triplicate. Control cells were incubated a without test sample and were incubated with or without DMSO. After the cells were incubated, the viable cell yield was determined using a colorimetric method [17,64].

In brief, after the end of the incubation period, the media were aspirated, and the crystal violet solution (1%) was added to each well for at least 30 min. The stain was removed, and the plates were rinsed using tap water until all of the excess stain was removed. Glacial acetic acid (30%) was then added to all of the wells and mixed thoroughly, and then the absorbances of the plates were measured after the plates had been gently shaken on a microplate reader (TECAN, Inc., Durham, NC, USA) using a test wavelength of 490 nm. All of the results were corrected for the background absorbance detected in the wells that did not have any added stain. Treated samples were compared to the control cells in the absence of the tested compounds.

All of the experiments were carried out in triplicate. The cytotoxic effect of each tested compound on the different cell lines was calculated. The optical density was measured with a microplate reader (SunRise, TECAN, Inc.) to determine the number of viable cells. The cell viability percentage was calculated as [(ODt/ODc)] × 100%, where ODt is the mean optical density of the wells treated with the tested sample, and ODc is the mean optical density of the untreated cells.

The relationship between the surviving cells and the drug concentration was plotted to determine the survival curve of each tumor cell line after treatment with the specified compound [64].

The cytotoxic concentration (CC_50_), the concentration required to cause toxic effects in 50% of the intact cells, was estimated from the graphic plots of the dose–response curve for each concentration using Graphpad Prism software (San Diego, CA, USA).

## 4. Conclusions

In conclusion, different physicochemical techniques were utilized to examine and confirm new Gd(III), Sm(III), and Nd(III) complexes from newly synthesized Schiff base. All of the complexes had the same molar ratio of 2:1 based on their stoichiometry (ligand: lanthanide ion) in a neutral state. Strong antifungal and antibacterial activities were reported for the newly synthesized Schiff base compound and its complexes. The antibacterial effect reveals that the metal complexes exhibit higher levels of antimicrobial activity than the free ligand. Using the complex phosphomolybdate method, the free ligand and its complexes were also found to have good antioxidant activity. In addition, a wide range of cytotoxic effects were observed for the free ligand and its complexes. Compounds such as these may have therapeutic uses, but more studies on the in vivo antioxidants, molecular studies, mechanisms of action, and animal studies are required to prove their safety and effectiveness.

## Figures and Tables

**Table 1:** Peak assignment in FTIR spectra (cm^−1^) of **L**, **La**, **Lb**, and **Lc**.

Compounds	υ(HC=N) Imine	γ(HC=N) Imine	υ(C=N) Thiazole	C-S-C Thiazole	C-O Furane	υH_2_O Lattice	υ(Ln-S)	υ(Ln-N)
**L**	1660	1016	1603	758	1057	-	-	-
**La**	1642	1045	1601	804	1060	3450	416	547
**Lb**	1646	1044	1601	805	1060	3477	428	563
**Lc**	1645	1034	1599	815	1060	3465	421	558

**Table 2:** Physicochemical properties of synthesized compounds.

Compound	Molecular Formula Molar Mass g Mol^−1^ (*m*/*z*)	Yield g (%)	m. p. (°C) Color	μeff	Λm: Molar Conductance S.L/mol	Elemental Analysis: Calculated (Found)			
						C (%)	H (%)	N (%)	S (%)
**L**	**C_16_H_14_N2O2S** **298.360** **(298.80)**	0.92 g 78.13%	145–146 (Orange Yellow)	-	-	64.41 (64.63)	4.73 (4.79)	9.39 (9.52)	10.75 (10.81)
**La**	**C_32_H_30_N_7_O_14_S_2_Gd** **957.997** **(958.77)**	0.70 g 87.20%	195–196 (Deep orange)	7.90	23	40.12 (40.29)	3.16 (3.20)	10.23 (10.51)	6.69 (6.80)
**Lb**	**C_32_H_30_N_7_O_14_S_2_Sm** **951.107** **(952.63)**	0.72 g 89.70%	210–211 (Pale orange)	1.88	25	40.41 (40.32)	3.18 (3.19)	10.31 (10.54)	6.74 (6.82)
**Lc**	**C_32_H_30_N_7_O_14_S_2_Nd** **944.989** **(947.69)**	0.65 g 80.97%	224–225 (Deep orange)	3.24	28	40.67 (40.91)	3.20 (3.26)	10.38 (10.59)	6.79 (6.72)

**Table 3:** Total antioxidant activity and DPPH radical scavenging activity of the Schiff base compound and its complexes.

Sample Code	TAC (mg GAE/g)	DPPH IC_50_ (µg/mL)
**L**	25.97 ± 2.38	478.52 ± 13.84
**La**	18.71 ± 3.41	570.16 ± 14.08
**Lb**	17.04 ± 1.91	505.82 ± 11.76
**Lc**	28.85 ± 3.32	410.18 ± 15.34
**Ascorbic acid**	66.08 ± 1.18	10.56 ± 0.78
**BHT**	71.82 ± 1.76	5.78 ± 0.27

BHT—butyl hydroxytoluene.

**Table 4:** Minimum inhibitory concentration in mm ± SD of Schiff base compound and its complexes.

Sample	Diameter of Inhibition Zones (mm)				
	L	La	Lb	Lc	Control
	Mean ± SD	Mean ± SD	Mean ± SD	Mean ± SD	Mean ± SD
**Fungi**					**Ketoconazole**
*Aspergillus fumigatus*	22.20 ± 0.56	17.01 ± 0.02	19.00 ± 0.30	23.13 ± 0.31	17.01 ± 0.02
*Candida albicans*	20.10 ± 0.40	20.03 ± 0.04	17.97 ± 0.35	18.78 ± 0.36	20.03 ± 0.04
**Gram-Positive Bacteria**					**Gentamycin**
*Staphylococcus aureus*	15.17 ± 0.55	24.01 ± 0.01	14.20 ± 0.36	18.07 ± 0.35	24.01 ± 0.01
*Bacillus subtilis*	20.10 ± 0.36	26.02 ± 0.03	18.10 ± 0.30	20.80 ± 0.52	26.02 ± 0.03
**Gram-Negative Bacteria**					**Gentamycin**
*Escherichia coli*	18.17 ± 0.40	29.90 ± 0.01	17.10 ± 0.36	22.60 ± 0.40	29.90 ± 0.01
*Proteus vulgaris*	16.97 ± 0.42	25.00 ± 0.01	14.00 ± 0.26	18.60 ± 0.26	25.00 ± 0.01

**Table 5:** The inhibitory effects (IC_50_%) of the Schiff base compound and its complexes against different types of cell lines.

Compound L µg/mL		Compound La µg/mL		Compound Lb µg/mL		Compound Lc µg/mL	
**HepG-2**	41.19 ± 6.70	**Huh-7**	164.72 ± 9.66	**HCT-116**	50.21 ± 5.03	**HepG-2**	42.18 ± 5.56
**MCF-7**	84.90 ± 6.98	**MDA-MB-231**	106.31 ± 5.97	**CACO2**	40.15 ± 0.31	**MCF-7**	55.42 ± 3.60
**PC-3**	58.81 ± 2.53	**A-549**	83.28 ± 2.36	**RD**	82.52 ± 10.16	**HEP-2**	105.23 ± 11.49
**WISH**	>500	**MRC-5**	>500	**WI-38**	>500	**HFB4**	>500

HepG-2: human hepatocellular carcinoma, MCF-7: human breast cancer cell line, PC-3: prostate carcinoma, WISH: human amnion (normal liver cells), Huh-7: human hepatocellular cancer cell line, MDA-MB231: human breast carcinoma, A-549: lung carcinoma, MRC-5: normal human lung fibroblast, HCT-116: human colon carcinoma, CACO2: human lung fibroblast normal cell line, RD: human muscle Rhabdomyosarcoma cell, WI-38: human lung fibroblast normal cells, HEP-2: larynx carcinoma cells, HFB4: human normal melanocytes.

## Data Availability

Data is contained within the article and supplementary material.

## Supplementary Materials

The following supporting information can be downloaded at: https://www.mdpi.com/article/10.3390/ph15040454/s1, Figure S1: 1H NMR spectrum of I in CAN, Figure S2: 13C NMR spectrum of I in CAN, Figure S3: FTIR spectra of **L**, **La**, **Lb**, and **Lc**, Figure S4: UV-vis spectra of **L**, **La**, **Lb**, and **Lc** in CAN, Figure S5: Mass spectrum of **L**, Figure S6: Mass spectrum of **La**, Figure S7: Mass spectrum of **Lb**, Figure S8: Mass spectrum of **Lc**, Figure S9: Thermogravimetric spectrum of **La**, Figure S10: Thermogravimetric spectrum of **Lb**, Figure S11: Thermogravimetric spectrum of **Lc.**

## References

[1] Xavier A., Srividhya N. (2014). Synthesis and study of Schiff base ligands. IOSR J. Appl. Chem..

[2] Badihian S., Shaygannejad V., Soleimani P., Mirmosayyeb O., Samee Z., Manouchehri N., Esmaeil N. (2018). Decreased serum levels of interleukin-35 among multiple sclerosis patients may be related to disease progression. J. Biol. Regul. Homeost. Agents.

[3] Buldurun K., Turan N., Savcı A., Çolak N. (2019). Synthesis, structural characterization and biological activities of metal (II) complexes with Schiff bases derived from 5-bromosalicylaldehyde: Ru (II) complexes transfer hydrogenation. J. Saudi Chem. Soc..

[4] Shaygan S., Pasdar H., Foroughifar N., Davallo M., Motiee F. (2018). Cobalt (II) complexes with Schiff base ligands derived from terephthalaldehyde and ortho-substituted anilines: Synthesis, characterization and antibacterial activity. Appl. Sci..

[5] Cotton S.A. (2005). Establishing coordination numbers for the lanthanides in simple complexes. Comptes Rendus Chim..

[6] Al Momani W.M., Taha Z.A., Ajlouni A.M., Shaqra Q.M.A., Al Zouby M. (2013). A study of in vitro antibacterial activity of lanthanides complexes with a tetradentate Schiff base ligand. Asian Pac. J. Trop. Biomed..

[7] Sagar Babu S.V., Krishna Rao K., Ill Lee Y. (2017). Synthesis, characterization, luminescence and DNA binding properties of Ln (III)-Schiff base family. J. Chil. Chem. Soc..

[8] Wang X., Yang Y.-L., Wang P., Li L., Fan R.-Q., Cao W.-W., Yang B., Wang H., Liu J.-Y. (2012). High efficiency co-sensitized solar cell based on luminescent lanthanide complexes with pyridine-2,6-dicarboxylic acid ligands. Dalton Trans..

[9] Wittig G., Schöllkopf U. (1954). Über Triphenyl-phosphin-methylene als olefinbildende Reagenzien I. Mitteil. Chem. Ber..

[10] Heck R.F., Nolley J.P. (1972). Palladium-catalyzed vinylic hydrogen substitution reactions with aryl, benzyl, and styryl halides. J. Org. Chem..

[11] Milstein D., Stille J. (1979). Palladium-catalyzed coupling of tetraorganotin compounds with aryl and benzyl halides. Synthetic utility and mechanism. J. Am. Chem. Soc..

[12] Anzini M., Chelini A., Mancini A., Cappelli A., Frosini M., Ricci L., Valoti M., Magistretti J., Castelli L., Giordani A. (2010). Synthesis and biological evaluation of amidine, guanidine, and thiourea derivatives of 2-amino (6-trifluoromethoxy) benzothiazole as neuroprotective agents potentially useful in brain diseases. J. Med. Chem..

[13] Kathiresan S., Annaraj J., Bhuvanesh N.S. (2017). Cu (II) and Ni (II) Complexes of Anthracene-Affixed Schiff Base: A Conflict between Covalent and Stacking Interactions with DNA Bases. ChemistrySelect.

[14] Song X.-Q., Wang Z.-G., Wang Y., Huang Y.-Y., Sun Y.-X., Ouyang Y., Xie C.-Z., Xu J.-Y. (2020). Syntheses, characterization, DNA/HSA binding ability and antitumor activities of a family of isostructural binuclear lanthanide complexes containing hydrazine Schiff base. J. Biomol. Struct. Dyn..

[15] Kafi-Ahmadi L., Marjani A.P. (2019). Mononuclear Schiff base complexes derived from 5-azophenylsalicylaldehyde with Co (ii), Ni (ii) ions: Synthesis, characterization, electrochemical study and antibacterial properties. S. Afr. J. Chem..

[16] Kaczmarek M.T., Zabiszak M., Nowak M., Jastrzab R. (2018). Lanthanides: Schiff base complexes, applications in cancer diagnosis, therapy, and antibacterial activity. Coord. Chem. Rev..

[17] El-Gammal O.A., Mohamed F.S., Rezk G.N., El-Bindary A.A. (2021). Structural characterization and biological activity of a new metal complexes based of Schiff base. J. Mol. Liq..

[18] Ejidike I.P., Ajibade P.A. (2015). Synthesis, characterization, antioxidant, and antibacterial studies of some metal (II) complexes of tetradentate schiff base ligand:(4E)-4-[(2-(E)-[1-(2, 4-dihydroxyphenyl) ethylidene] aminoethyl) imino] pentan-2-one. Bioinorg. Chem. Appl..

[19] Keshavayya J., Pandurangappa M., Ravi B. (2018). Synthesis, characterization and electrochemical investigations of azo dyes derived from 2-Amino-6-ethoxybenzothiazole. Chem. Data Collect..

[20] Ashashi N.A., Kumar M., Ul Nisa Z., Frontera A., Sahoo S.C., Sheikh H.N. (2021). Solvothermal self-assembly of three lanthanide (III)-succinates: Crystal structure, topological analysis and DFT calculations on water channel. J. Mol. Struct..

[21] Abou-Melha K., Faruk H. (2008). Bimetallic complexes of Schiff base bis-[4-hydroxycuomarin-3-yl]-1N,5N-thiocarbohydrazone as a potentially dibasic pentadentate ligand. Synthesis, spectral, and antimicrobial properties. J. Iran. Chem. Soc..

[22] Pilichos E., Font-Bardia M., Escuer A., Mayans J. (2021). Structural and magnetic studies of mononuclear lanthanide complexes derived from N-rich chiral Schiff bases. Dalton Trans..

[23] Capan A., Uruş S., Sönmez M. (2018). Ru (III), Cr (III), Fe (III) complexes of Schiff base ligands bearing phenoxy Groups: Application as catalysts in the synthesis of vitamin K3. J. Saudi Chem. Soc..

[24] Kaya İ., Demir H.Ö., Vilayetoğlu A.R. (2002). The synthesis and characterisation of planar oligophenol with Schiff base substitute. Synth. Met..

[25] Etaiw S.E.H., Abd El-Aziz D.M., Abd El-Zaher E.H., Ali E.A. (2011). Synthesis, spectral, antimicrobial and antitumor assessment of Schiff base derived from 2-aminobenzothiazole and its transition metal complexes. Spectrochim. Acta Part A Mol. Biomol. Spectrosc..

[26] Mabkhot Y.N., Al-Showiman S.S., Barakat A., Soliman S., Kheder N.A., Alharbi M.M., Asayari A., Muhsinah A.B., Ullah A., Badshah S.L. (2019). Computational studies of 2-(4-oxo-3-phenylthiazolidin-2-ylidene) malononitrile. BMC Chem..

[27] Dehouche Z., Klassen T., Oelerich W., Goyette J., Bose T., Schulz R. (2002). Cycling and thermal stability of nanostructured MgH_2_–Cr_2_O_3_ composite for hydrogen storage. J. Alloy. Compd..

[28] Dubey R., Dubey U., Mishra C. (2008). Synthesis and physicochemical characterization of some Schiff base complexes of chromium (III). Indian J. Chem. Sect. A.

[29] Bhowon M.G., Li Kam Wah H., Dosieah A., Ridana M., Ramalingum O., Lacour D. (2004). Synthesis, characterization, and catalytic activity of metal Schiff base complexes derived from pyrrole-2-carboxaldehyde. Synth. React. Inorg. Met.-Org. Chem..

[30] Abdul-Ghani A.J., Khaleel A. (2009). Synthesis and characterization of new schiff bases derived from N (1)-substituted isatin with dithiooxamide and their co (II), Ni (II), Cu (II), Pd (II), and Pt (IV) complexes. Bioinorg. Chem. Appl..

[31] Yao L., Su C., Qi L., Liu C., Hu Y., Zhao H. (1999). The substituent structures and characteristic infrared spectra of alpha-furan esters. Guang Pu Xue Yu Guang Pu Fen Xi = Guang Pu.

[32] Eltayeb M., Li S., Okoye P.U., Wang S. (2021). Carbodiimide-Assisted Synthesis of High Purity Bis (cyclic carbonate) Under Atmospheric Conditions for Preparation of Non-Isocyanate Polyurethane. J. Polym. Environ..

[33] Marotta A., Ambrogi V., Cerruti P., Mija A. (2018). Green approaches in the synthesis of furan-based diepoxy monomers. RSC Adv..

[34] Naeimi H., Salimi F., Rabiei K. (2006). Mild and convenient one pot synthesis of Schiff bases in the presence of P_2_O_5_/Al_2_O_3_ as new catalyst under solvent-free conditions. J. Mol. Catal. A Chem..

[35] Arora K., Chaubey A., Singhal R., Singh R.P., Pandey M., Samanta S., Malhotra B., Chand S. (2006). Application of electrochemically prepared polypyrrole–polyvinyl sulphonate films to DNA biosensor. Biosens. Bioelectron..

[36] Zheng W., Hu J., Han Z., Wang Z., Zheng Z., Langer J., Economy J. (2015). Synthesis of porous carbon fibers with strong anion exchange functional groups. Chem. Commun..

[37] Manna S., Mistri S., Bhunia A., Paul A., Zangrando E., Manna S.C. (2017). Manganese (IV) complex with a polydentate Schiff base ligand: Synthesis, crystal structure, TDDFT calculation, electronic absorption and EPR spectral study. J. Coord. Chem..

[38] Sinha A., Bala M. (1991). Coordination behavior of herbicidal Schiff bases derived from 2-Amino-6-ethoxybenzothiazole towards copper (II). Asian J. Chem..

[39] Tsantis S.T., Zagoraiou E., Savvidou A., Raptopoulou C.P., Psycharis V., Szyrwiel L., Hołyńska M., Perlepes S.P. (2016). Binding of oxime group to uranyl ion. Dalton Trans..

[40] Sarıoğlu A.O., Yalçın Ş.P., Ceylan Ü., Aygün M., Kırpık H., Sönmez M. (2020). Photoluminescence properties of samarium (III)-based complexes: Synthesis, characterization and single crystal X-ray. J. Lumin..

[41] Godlewska P., Hanuza J., Kucharska E., Solarz P., Roszak S., Kaczmarek S.M., Leniec G., Ptak M., Kopacz M., Hermanowicz K. (2020). Optical and magnetic properties of lanthanide (III) complexes with quercetin-5′-sulfonic acid in the solid state and silica glass. J. Mol. Struct..

[42] Huidrom B., Devi N.R., Singh T.D., Singh N.R. (2012). Studies on the complexation of neodymium (III) ion with 1,2,4-1H-triazole and 1,2,3-benzotriazole in absence and presence of calcium (II) ion in aqueous and some selected different aquated organic solvents by an absorption spectroscopy involving 4f–4f transitions. Spectrochim. Acta Part A Mol. Biomol. Spectrosc..

[43] Irfanullah M., Iftikhar K. (2009). New dinuclear lanthanide (III) complexes based on 6,6,7,7,8,8,8-heptafluoro-2,2-dimethyl-3,5-octanedione and 2,2′-bipyrimidine. Inorg. Chem. Commun..

[44] Abu-Yamin A.-A. (2022). Synthesis, characterization, and crystal structure of LnIII–(1E, 2E)-3-(furan-2-yl)-N-(4H-1,2,4-triazol-4-yl) prop-2-en-1-imine. J. Coord. Chem..

[45] Prabhumirashi L., Khoje J. (2002). TGA and DTA studies on en and tmn complexes of Cu (II) chloride, nitrate, sulphate, acetate and oxalate. Thermochim. Acta.

[46] Mosmann T. (1983). Rapid colorimetric assay for cellular growth and survival: Application to proliferation and cytotoxicity assays. J. Immunol. Methods.

[47] Rao N.S., Mishra D., Maurya R., Rao N.N. (1995). Synthesis and Characterisation of Some Novel CIS-Dioxo-Molybdenum (VI) Complexes of Schiff Bases Derived from Salicylaldehyde. Synth. React. Inorg. Met.-Org. Chem..

[48] Alkreathy H.M., Esmat A. (2022). Lycorine Ameliorates Thioacetamide-Induced Hepatic Fibrosis in Rats: Emphasis on Antioxidant, Anti-Inflammatory, and STAT3 Inhibition Effects. Pharmaceuticals.

[49] Prieto P., Pineda M., Aguilar M. (1999). Spectrophotometric quantitation of antioxidant capacity through the formation of a phosphomolybdenum complex: Specific application to the determination of vitamin E. Anal. Biochem..

[50] Yen G.C., Duh P.D. (1994). Scavenging effect of methanolic extracts of peanut hulls on free-radical and active-oxygen species. J. Agric. Food Chem..

[51] Saghir S.A.M., Sadikun A., Al-Suede F.S., MSA Majid A., Murugaiyah V. (2016). Antihyperlipidemic, antioxidant and cytotoxic activities of methanolic and aqueous extracts of different parts of star fruit. Curr. Pharm. Biotechnol..

[52] Abo-Ashour M.F., Eldehna W.M., George R.F., Abdel-Aziz M.M., Elaasser M.M., Gawad N.M.A., Gupta A., Bhakta S., Abou-Seri S.M. (2018). Novel indole-thiazolidinone conjugates: Design, synthesis and whole-cell phenotypic evaluation as a novel class of antimicrobial agents. Eur. J. Med. Chem..

[53] Ho P., Chow K., Tse H., Cheng V. (2012). Effect of applying the new clinical and laboratory standards. Int. J. Antimicrob. Agents.

[54] Ibrahim H.S., Eldehna W.M., Abdel-Aziz H.A., Elaasser M.M., Abdel-Aziz M.M. (2014). Improvement of antibacterial activity of some sulfa drugs through linkage to certain phthalazin-1(2H)-one scaffolds. Eur. J. Med. Chem..

[55] Kumaravel G., Utthra P.P., Raman N. (2018). Exploiting the biological efficacy of benzimidazole based Schiff base complexes with l-Histidine as a co-ligand: Combined molecular docking, DNA interaction, antimicrobial and cytotoxic studies. Bioorg. Chem..

[56] Fetoh A., Asla K.A., El-Sherif A.A., El-Didamony H., El-Reash G.M.A. (2019). Synthesis, structural characterization, thermogravimetric, molecular modelling and biological studies of Co (II) and Ni (II) Schiff bases complexes. J. Mol. Struct..

[57] Gomha S.M., Riyadh S.M., Mahmmoud E.A. (2015). Synthesis and anticancer activities of thiazoles,1,3-thiazines, and thiazolidine using chitosan-grafted-poly (vinylpyridine) as basic catalyst. Heterocycles Int. J. Rev. Commun. Heterocycl. Chem..

[58] Ejidike I.P., Ajibade P.A. (2016). Synthesis, characterization, anticancer, and antioxidant studies of Ru (III) complexes of monobasic tridentate Schiff bases. Bioinorg. Chem. Appl..

[59] Kumar M., Padmini T., Ponnuvel K. (2017). Synthesis, characterization and antioxidant activities of Schiff bases are of cholesterol. J. Saudi Chem. Soc..

[60] Jarrahpour A., Motamedifar M., Pakshir K., Hadi N., Zarei M. (2004). Synthesis of novel azo Schiff bases and their antibacterial and antifungal activities. Molecules.

[61] Ahmed H., Mahmoud W.H., Al-Akraa Dr I.M., Mohamed G.G. (2020). Preparation, Characterization and Biological Activity of Novel Schiff Base Complexes based on La, Er and Yb Metal Ions. ARPN J. Eng. Appl. Sci..

[62] Elsonbati A., Diab M.A., Mohamed G., Morgan S. (2021). Preparation, Characterization and Biological Activity Screening on Some Metal Complexes Based of Schiff Base Ligand. Egypt. J. Chem..

[63] Uddin N., Rashid F., Ali S., Tirmizi S.A., Ahmad I., Zaib S., Zubair M., Diaconescu P.L., Tahir M.N., Iqbal J. (2020). Synthesis, characterization, and anticancer activity of Schiff bases. J. Biomol. Struct. Dyn..

[64] Poonia K., Siddiqui S., Arshad M., Kumar D. (2016). In Vitro anticancer activities of Schiff base and its lanthanum complex. Spectrochim. Acta Part A Mol. Biomol. Spectrosc..

