# Supplementary material for: Synthesis, Characterization and Biological Activities of New Schiff Base Compound and Its Lanthanide Complexes
Source: Pharmaceuticals (Basel). 2022 Apr 7;15(4):454. doi: 10.3390/ph15040454 (PMC9027428; doi:10.3390/ph15040454)
Supplement: Supplementary file 1 [file pharmaceuticals-15-00454-s001.zip › pharmaceuticals-1641556-supplementary.pdf]

Synthesis, Characterization and Biological Activities of New Schiff Base Compound and Its Lanthanide Complexes

Abdel-Aziz Abu-Yamin [1,]*, Maisa Siddiq Abduh [2,3,]*, Sultan Ayesh Mohammed Saghir [4] and Naif Al-Gabri [5,6]

[1]Department of Chemistry, Al-Hussein Bin Talal University, Ma’an, 71111 Jordan
[2]Department of Medical Laboratory Sciences, Faculty of Applied Medical Sciences, King Abdulaziz University, Jeddah 21589, Saudi Arabia
[3]Center of Excellence in Genomic Medicine Research, King Abdulaziz University, Jeddah 22252, Saudi Arabia
[4]Department of Medical Analysis. Princess Aisha Bint Al-Hussein College of Nursing and Medical Sciences, Al-Hussein Bin Talal University, Ma’an 71111, Jordan; Sultan.S.Ayesh@ahu.edu.jo
[5]Veterinary Department, Faculty of Agriculture and Veterinary Medicine, Thamar University, Dhamar 2153, Yemen; naifaljabry2014@gmail.com
[6]Laboratory of Salam Veterinary Group, Buraydah, Al-Qassim 51911, Saudi Arabia

- Correspondence: abuyamin@ahu.edu.jo (A.-A.A.-Y.); mabdoh@kau.edu.sa (M.S.A.); Tel.: +00962-772229226 (A.-A.A.-Y.); Tel: +00966-568026868 (M.S.A.)

Contents:

Figure	Title	page
Figure S1	1H NMR spectrum of L in ACN.	2
Figure S2	13C NMR spectrum of L in ACN.	2
Figure S3	FTIR spectra of L, La, Lb and Lc.	3
Figure S4	UV-vis spectra of L, La, Lb and Lc in ACN.	3
Figure S5	Mass spectrum of L in ACN.	4
Figure S6	Mass spectrum of La in ACN.	4
Figure S7	Mass spectrum of Lb in ACN.	4
Figure S8	Mass spectrum of Lc in ACN.	5
Figure S9	Thermogravimetric spectrum of La	5
Figure S10	Thermogravimetric spectrum of Lb	6
Figure S11	Thermogravimetric spectrum of Lc	6

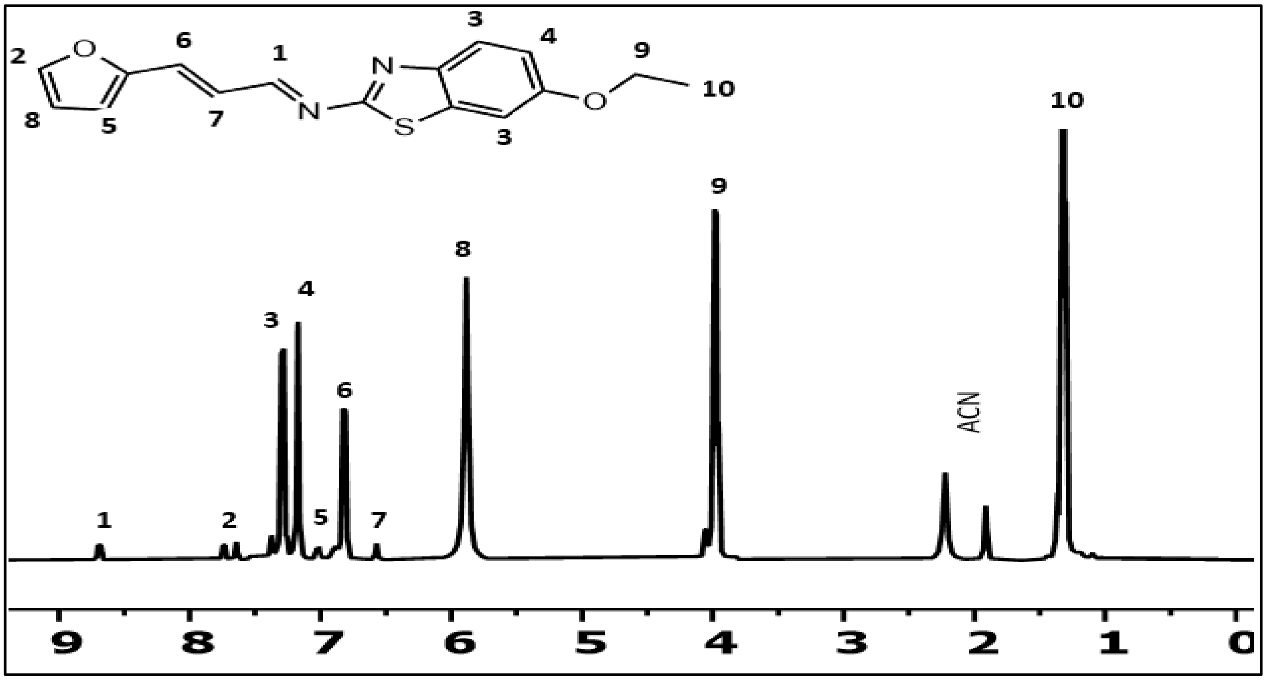

Figure S1: ^{1}H NMR spectrum of **L** in ACN**.**

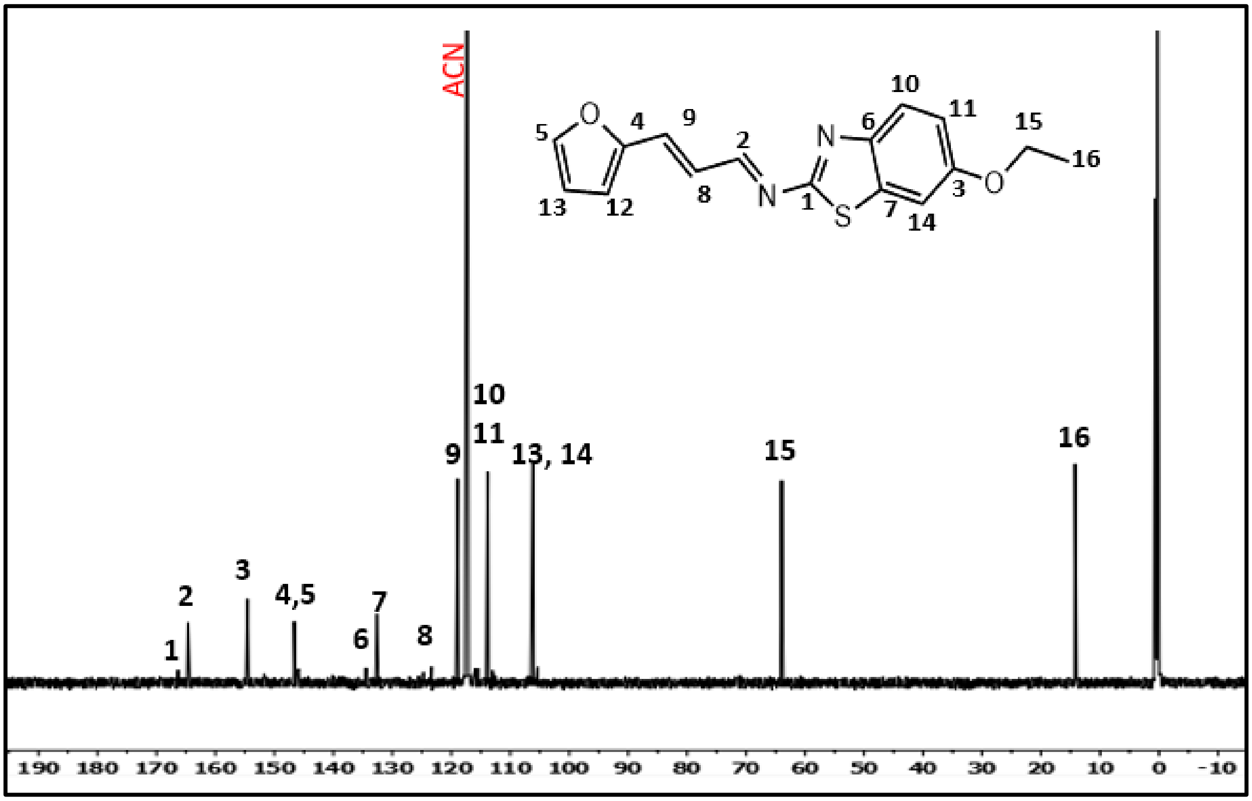

Figure S2: ^{13}C NMR spectrum of **L** in ACN.

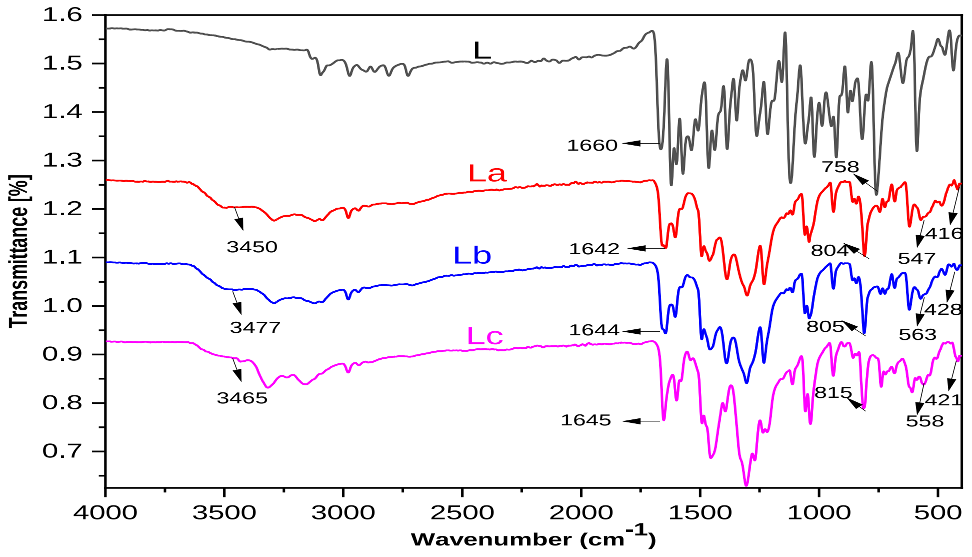

Figure S3: FTIR spectra of **L**, $\mathbf{L_a}$, $\mathbf{L_b}$ and $\mathbf{L_c}$.

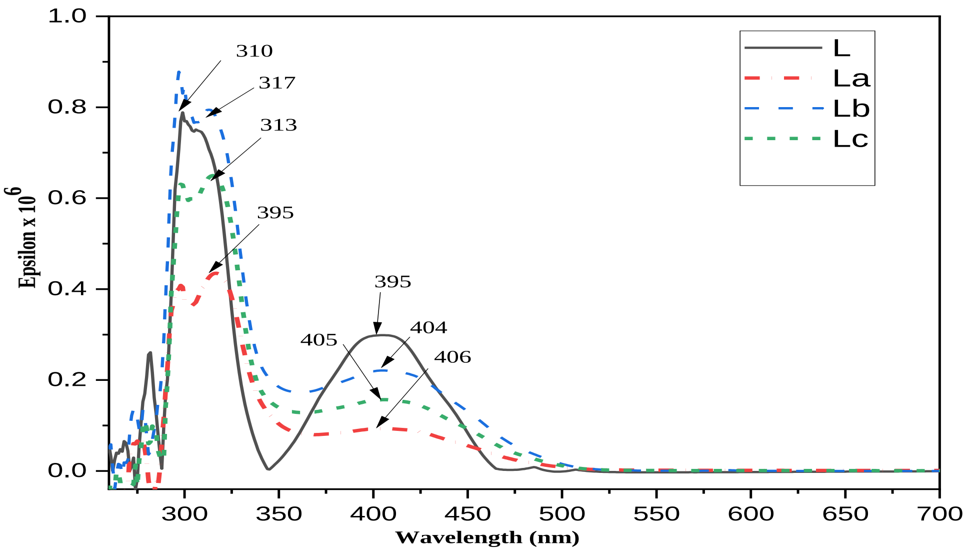

Figure S4: UV-vis spectra of **L,** $\mathbf{L_a}$**,** $\mathbf{L_b}$ and $\mathbf{L_c}$ in ACN.

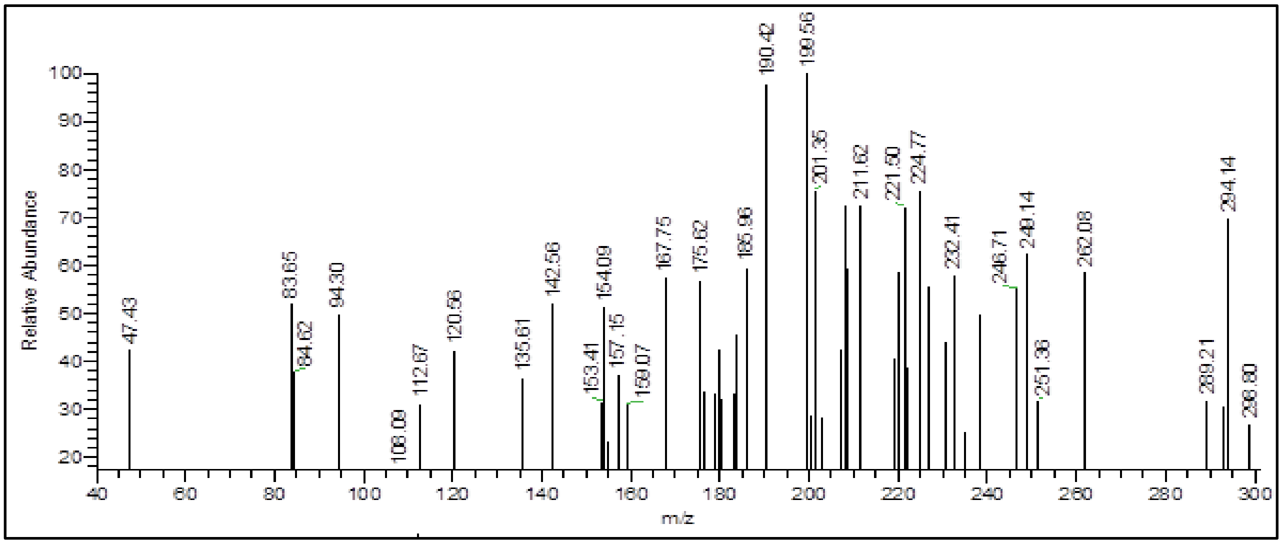

Figure S5: Mass spectrum of **L**.

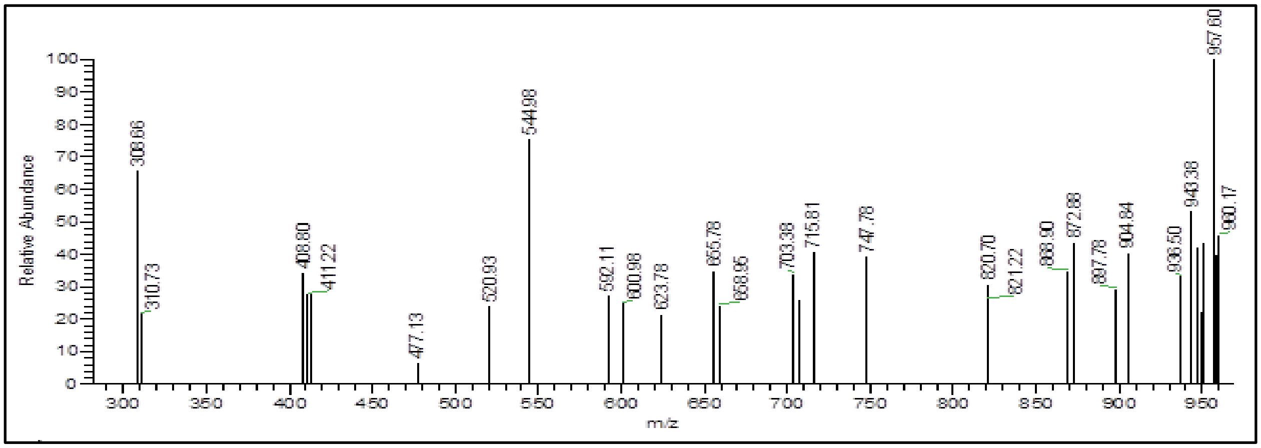

Figure S6: Mass spectrum of $\mathbf{L_a}$.

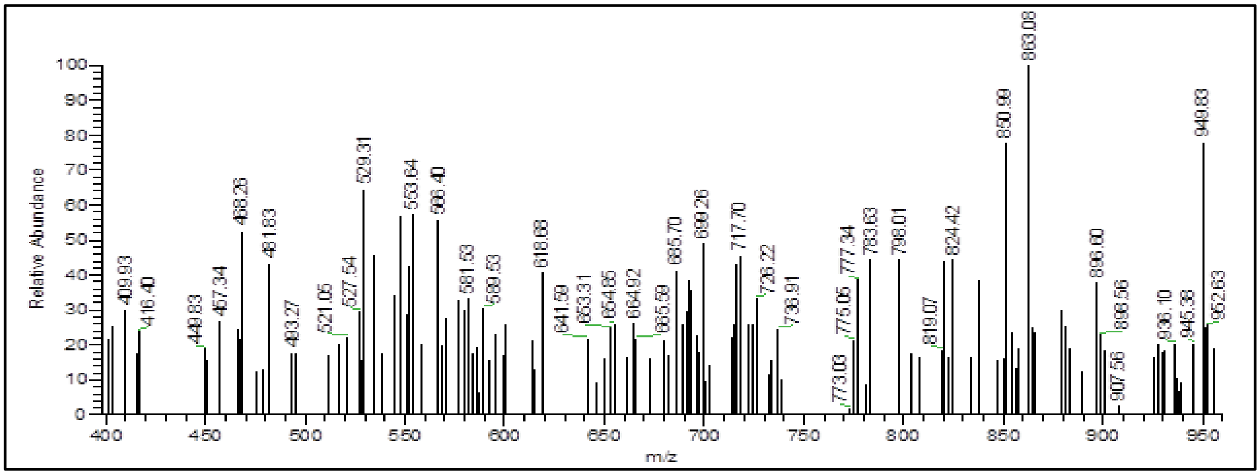

Figure S7: Mass spectrum of $\mathbf{L_b}$.

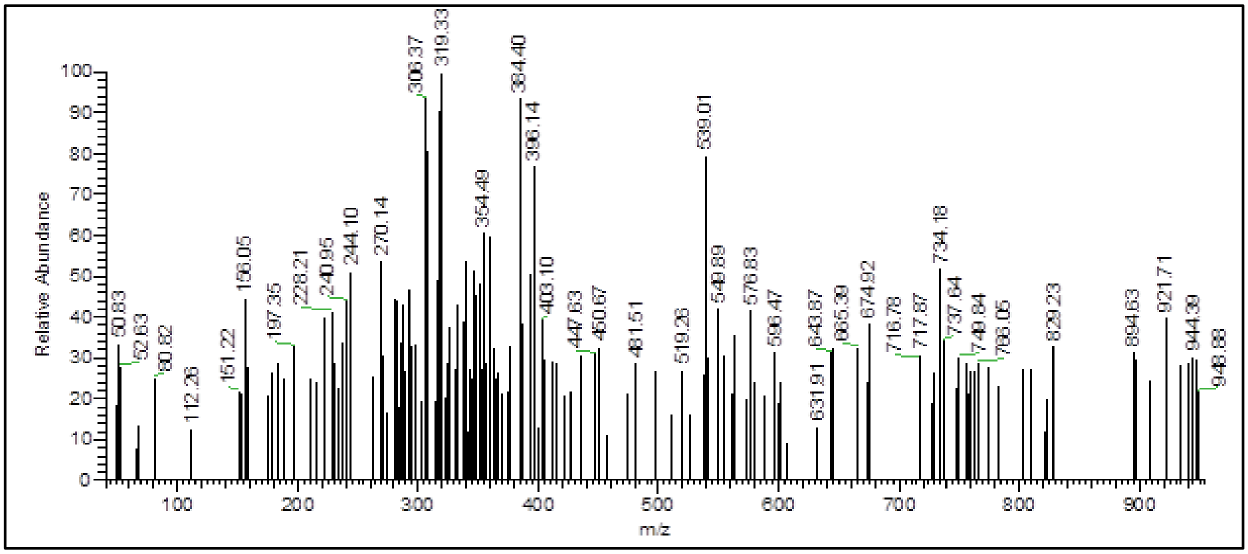

Figure S8: Mass spectrum of $\mathbf{L_c}$.

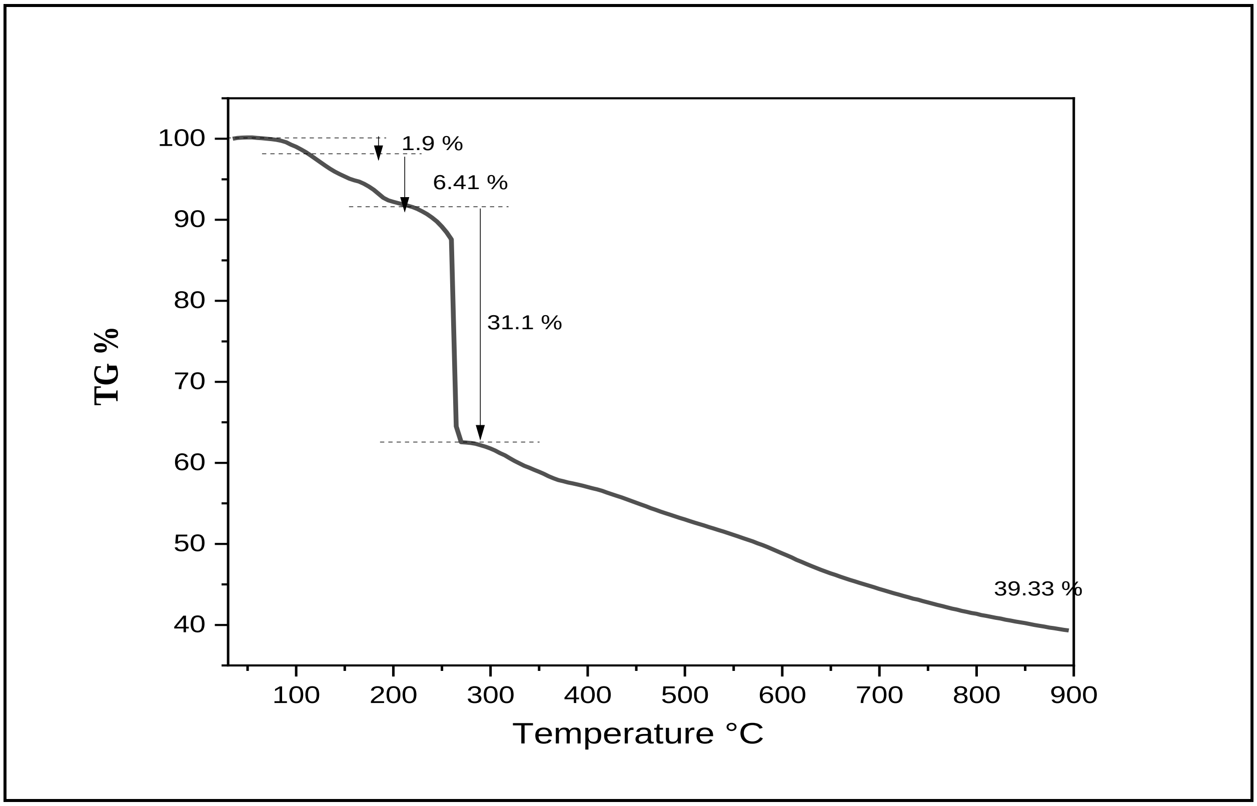

Figure S9: Thermogravimetric spectrum of $\mathbf{L_a}$

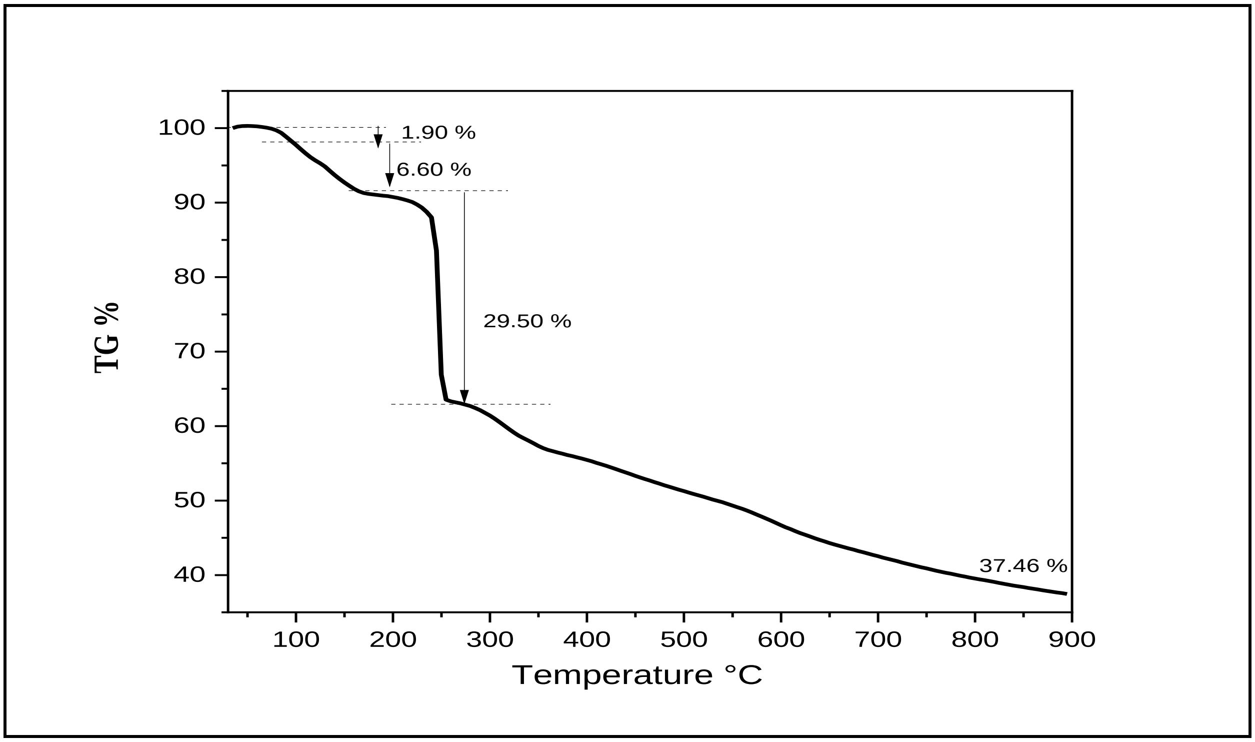

Figure S10: Thermogravimetric spectrum of $\mathbf{L_b}$

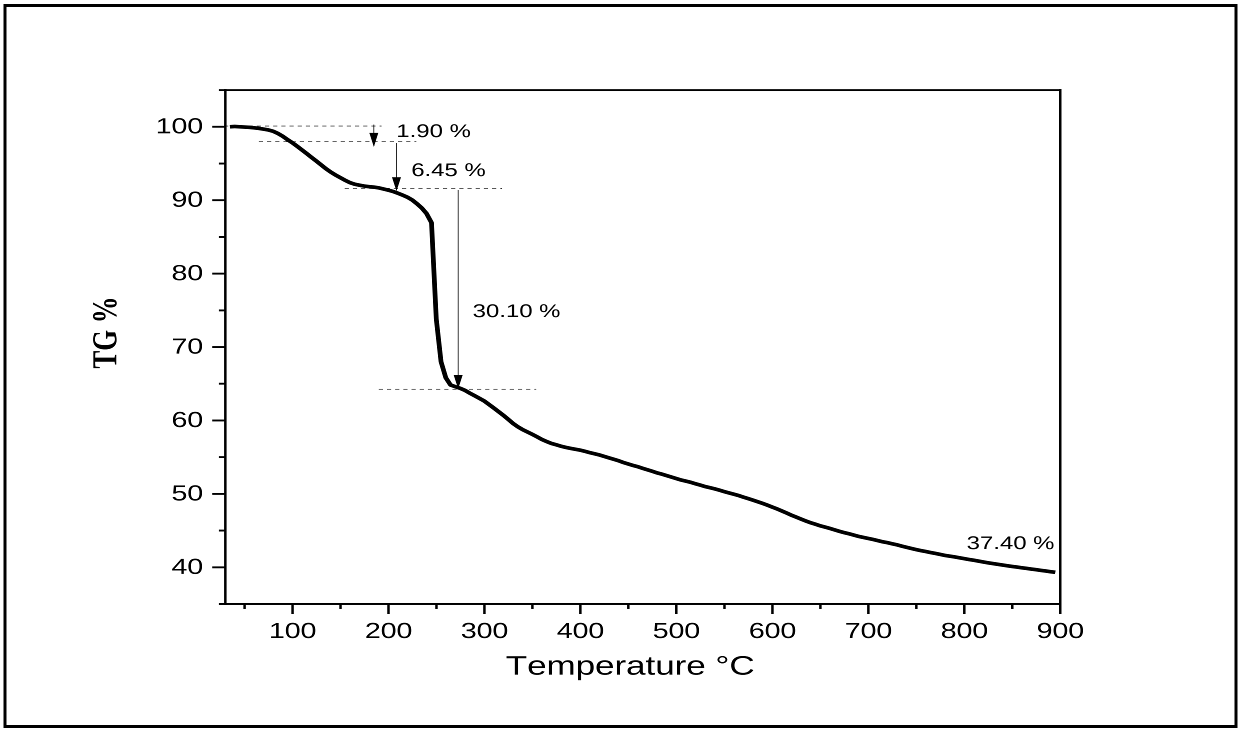

Figure S11: Thermogravimetric spectrum of $\mathbf{L_c}$
